# Antibiotic Resistance in Urine Culture Isolates from Hospitalized Children Under Five Years with Urinary Tract Infection: A Comparison of the 2019–2020 and 2024–2025 Periods

**DOI:** 10.3390/antibiotics15101031

**Published:** 2026-10-16

**Authors:** Elife Çapan Gün, Şeyma Şimşirgil Kara, Yasemin Akın

**Affiliations:** 1 Department of Pediatrics, Kartal Dr. Lütfi Kırdar City Hospital, Istanbul 34890, Türkiye; elife_capan@hotmail.com (E.Ç.G.); jasminetr@hotmail.com (Y.A.); 2 Faculty of Health Sciences, Sinop University, Sinop 57000, Türkiye

**Keywords:** urinary tract infection, antibiotic resistance, children, *Escherichia coli*, *Klebsiella pneumoniae*

## Abstract

**Background/Objectives:** We evaluated changes in antibiotic resistance among urine culture isolates from children under five years hospitalized with urinary tract infection (UTI) at a single center in 2019–2020 and 2024–2025. **Methods:** This retrospective, repeated cross-sectional study included 498 children and analyzed 359 isolates of *Escherichia coli*, *Klebsiella pneumoniae*, *Pseudomonas aeruginosa*, and *Proteus* spp. As second-line agents were tested selectively, primary outcomes (defined post hoc) were resistance to a core panel of agents tested in at least 90% of isolates and core-panel multidrug resistance (MDR) in Enterobacterales; other comparisons were exploratory. Fisher’s exact test, Benjamini–Hochberg correction, and adjusted Firth regression were used. **Results:** Second-line agents were tested mainly in cefuroxime-resistant isolates (95% vs. 7% for ceftazidime in *E. coli*), biasing resistance estimates. In the core panel, no difference was significant in *E. coli*, and the decrease in cefuroxime resistance in *K. pneumoniae* did not survive correction. Core-panel MDR was lower in 2024–2025 (51.9% vs. 38.7%; *p* = 0.019; adjusted OR 0.56, 95% CI 0.35–0.88); full-category MDR was not (68.4% vs. 58.3%; *p* = 0.067). **Conclusions:** Differences may reflect testing practices and case mix rather than true changes in resistance. Findings apply only to hospitalized children under five years at this center and require confirmation.

## 1. Introduction

Urinary tract infections (UTIs) are among the most common bacterial infections in childhood and are the underlying cause of fever in a substantial proportion of children [1]. A meta-analysis reported that the prevalence of childhood UTI varies with age, sex, and circumcision status [2]. In children under five years of age, UTIs cause acute morbidity and can lead to long-term complications, such as vesicoureteral reflux (VUR), renal scarring, and chronic kidney disease [1].

Selecting an appropriate empirical antibiotic is critical for treatment success and for preventing complications. However, the global rise in antimicrobial resistance makes empirical treatment increasingly difficult. *Escherichia coli* is the most common cause of pediatric UTI [3]; other Gram-negative bacteria, such as *Klebsiella pneumoniae*, *Proteus* species, and *Pseudomonas aeruginosa*, are also important causes, particularly in children with urinary tract anomalies or recurrent UTI [4].

Recent multicenter and nationwide studies have reported rising resistance to third-generation cephalosporins among pediatric uropathogens. In South Korea, cefotaxime resistance in children younger than 24 months rose from approximately 10% in the early 2010s to over 30% after 2020 [5]. In Israel, the proportion of extended-spectrum β-lactamase (ESBL)-producing *E. coli* rose from 6.1% to 25.4% between 2007 and 2021 [6], and U.S. surveillance reported a smaller increase between 2014 and 2023 [3]. ESBL status was not available in our dataset; our analysis therefore addresses phenotypic resistance to cephalosporins and other agents.

Studies from Türkiye also report high resistance, particularly to ampicillin, trimethoprim-sulfamethoxazole (TMP-SMX), and first- and second-generation cephalosporins [7,8]. Data from 2020 onward are similar. In a retrospective study of 2753 outpatient children in Adıyaman (2020–2024), *E. coli* resistance was 67.4% for ampicillin, 45.3% for cefixime, and about 33% for TMP-SMX [9]. In 66 *E. coli* isolates from a tertiary hospital in Istanbul (2018–2021), ampicillin resistance was 84.8%, and 33 isolates (50%) were ESBL-positive [10]. However, because resistance rates vary by region and over time, each center should periodically review its own resistance data and update its empirical treatment protocols accordingly [11].

The aim of this study was to compare the antibiotic resistance of urine isolates from children under five years of age who were hospitalized with UTI at a single center in 2019–2020 and 2024–2025 and thereby to inform current empirical treatment approaches. Because second-line agents were tested selectively, we defined the primary outcomes during revision, after reviewing testing coverage: resistance to a core panel of agents tested in at least 90% of isolates and core-panel multidrug resistance in Enterobacterales. All other comparisons were exploratory.

## 2. Results

### 2.1. Demographic, Clinical, and Imaging Characteristics

**Figure 1:**
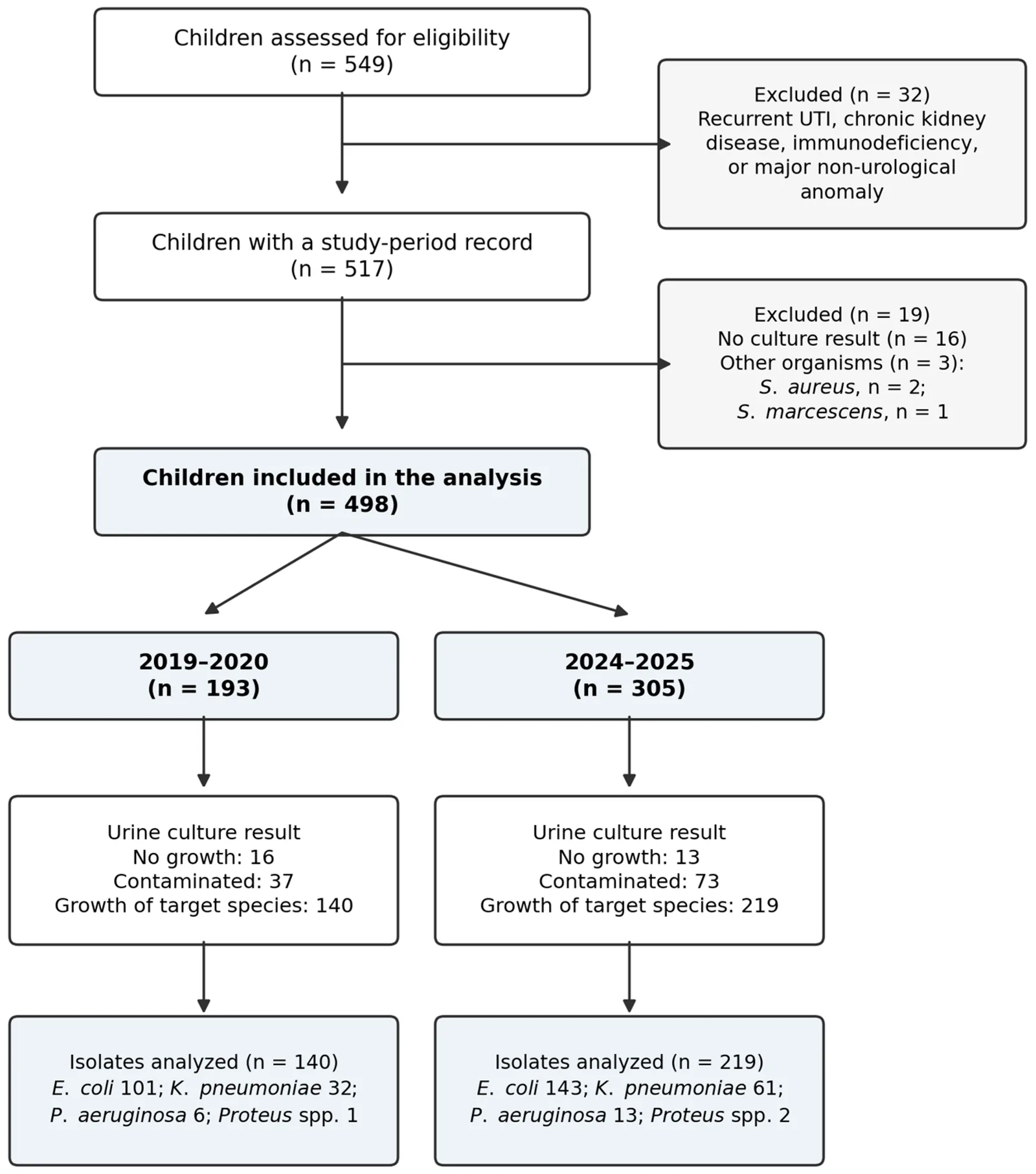
Flow diagram of the study population. UTI, urinary tract infection. Target species: *E. coli*, *K. pneumoniae*, *P. aeruginosa*, and *Proteus* spp.

A total of 498 children hospitalized with a clinical diagnosis of a first UTI episode were included: 193 in 2019–2020 and 305 in 2024–2025 (Figure 1 and Table 1). Of these, 359 had growth of one of the four target species and formed the culture-positive population for the resistance analyses. The proportion of girls was 62.2% in 2019–2020 and decreased to 46.2% in 2024–2025 (*p* < 0.001). Age did not differ significantly between periods (*p* = 0.695). The VUR rate decreased from 6.2% in 2019–2020 to 0.0% in 2024–2025 (*p* < 0.001); however, VCUG was performed about 3 weeks after the infection in 2019–2020 and about 12 weeks after it in 2024–2025. The proportion of children with structural anomalies or urolithiasis on ultrasonography rose from 12.0% (23/191) in 2019–2020 to 21.3% (65/305) in 2024–2025 (*p* = 0.009). Three children in 2024–2025 had infection-related findings (cystitis or pyelonephritis), which were not counted as anomalies. Hydronephrosis was the most common finding in both periods. The proportion of abnormal DMSA results, length of hospital stay, and urine culture growth status did not differ significantly between periods (*p* = 0.162, *p* = 0.409, and *p* = 0.105, respectively). The distribution of isolated species also did not differ (*p* = 0.601); *E. coli* was the most frequent pathogen in both periods (72.1% in 2019–2020 and 65.3% in 2024–2025), followed by *K. pneumoniae*.

### 2.2. Antibiotic Resistance in Proteus spp. Isolates

Only three *Proteus* isolates were identified (one in 2019–2020 and two in 2024–2025), and they were recorded at genus level only; no inferential comparison was performed. The 2019–2020 isolate and one 2024–2025 isolate were susceptible to all agents tested. The other 2024–2025 isolate was resistant to ampicillin, amoxicillin-clavulanate, cefuroxime, ceftriaxone, ceftazidime, cefixime, ciprofloxacin, and TMP-SMX, but susceptible to piperacillin-tazobactam, meropenem, ertapenem, and cefepime; it was classified as multidrug-resistant.

### 2.3. Antibiotic Resistance in Pseudomonas aeruginosa Isolates

In *P. aeruginosa* isolates (Table 2; 6 isolates in 2019–2020 and 13 in 2024–2025), resistance was not detected, except for imipenem in one of three tested isolates in 2019–2020 (0 of 13 in 2024–2025). Because of the small numbers, confidence intervals are wide (upper limits, 22.8–65.8%), and no inferential comparison was performed.

### 2.4. Antibiotic Resistance in Klebsiella pneumoniae Isolates

In *K. pneumoniae* isolates (Table 3), cefuroxime was the only core-panel agent with a nominal difference; resistance decreased from 71.9% to 49.2% (difference, −22.7 percentage points; 95% CI, −40.3 to −1.5; *p* = 0.047; *q* = 0.357 within the core family). Gentamicin (40.6% vs. 21.7%; *p* = 0.087), amoxicillin-clavulanate, and TMP-SMX did not differ. Ampicillin resistance was 100% in both periods, reflecting intrinsic resistance. Among exploratory agents, nominal differences were observed for ertapenem (64.7% vs. 25.9%; *p* = 0.015), cefixime (80.0% vs. 49.0%; *p* = 0.013), and amikacin (40.0% vs. 9.5%; *p* = 0.046), but none remained significant after Benjamini–Hochberg correction within the exploratory family (all *q* ≥ 0.058; Table S1). Agents tested in fewer than 10 isolates in either period (ciprofloxacin, imipenem, and cefepime) were not compared.

### 2.5. Antibiotic Resistance in Escherichia coli Isolates

In *E. coli* (Table 4), no core-panel agent differed significantly between periods: ampicillin, 81.2% vs. 72.6% (*p* = 0.158); cefuroxime, 66.0% vs. 56.0% (*p* = 0.142); gentamicin, 9.2% vs. 10.0%; amoxicillin-clavulanate, 45.3% vs. 37.4%; and TMP-SMX, 34.7% vs. 31.4% (all *q* ≥ 0.357). Among exploratory agents, nominal decreases were seen for ceftazidime (89.6% vs. 75.6%; *p* = 0.033), ceftriaxone (89.2% vs. 71.1%; *p* = 0.009), and cefixime (79.7% vs. 60.6%; *p* = 0.007), but none remained significant after correction (*q* ≥ 0.058; Table S1). These agents were tested selectively (Section 2.6).

### 2.6. Testing Coverage and Selective Testing

Susceptibility testing was not uniform across isolates (Table S2). Ampicillin, cefuroxime, gentamicin, amoxicillin-clavulanate, and TMP-SMX were tested in at least 90% of isolates in both periods and formed the core panel. All other agents were tested in 1–84% of isolates, and coverage differed between periods; for example, ciprofloxacin was tested in 15.6% versus 57.4%, and cefepime in 9.4% versus 41.0%, of *K. pneumoniae* isolates.

Second-line agents were tested predominantly in isolates that were already cefuroxime-resistant (Table S4). Among *E. coli* isolates, 95% of those tested for ceftazidime were cefuroxime-resistant, versus 7% of those not tested (*p* < 0.001); the corresponding proportions were 89% versus 10% for ceftriaxone, 94% versus 46% for ertapenem, and 88% versus 44% for piperacillin-tazobactam. In *K. pneumoniae*, the proportions were 95% versus 24% for ertapenem, 91% versus 36% for amikacin, and 80% versus 20% for piperacillin-tazobactam. This indicates cascade testing: resistance proportions for these agents describe pre-selected, cefuroxime-resistant isolates rather than all isolates, and differences between periods may reflect changes in testing coverage.

Because untested isolates may not be representative, we also estimated resistance to third-generation cephalosporins (ceftazidime or ceftriaxone) among all isolates of each species (Table S5). Documented resistance among all isolates assumes that untested isolates were susceptible and is therefore a lower bound. For *E. coli*, it was 66.3% (67/101) in 2019–2020 and 55.2% (79/143) in 2024–2025 (difference, −11.1 percentage points; 95% CI, −22.9 to +1.4; *p* = 0.087). For *K. pneumoniae*, it was 68.8% (22/32) and 49.2% (30/61) (difference, −19.6 percentage points; 95% CI, −37.6 to +1.6; *p* = 0.082). All but one of the untested isolates were cefuroxime-susceptible (cefuroxime was tested in 99% of isolates), so counting the single untested cefuroxime-resistant isolate as resistant would raise these estimates by at most 0.7 percentage points. This relies on the plausible but unverified assumption that cefuroxime-susceptible isolates are susceptible to third-generation cephalosporins. Without that assumption, the worst-case upper bounds, in which all untested isolates are resistant, are 97.0% and 88.1% for *E. coli* and 93.8% and 85.2% for *K. pneumoniae*; these bounds are uninformative. The estimates are lower than the 71–90% observed among tested *E. coli* isolates (Table 4), but the between-period differences are not statistically significant and are exploratory. ESBL production could not be assessed because confirmatory testing was not available.

Carbapenem results in *K. pneumoniae* were discordant. Among 32 isolates tested for both ertapenem and meropenem, 11 of 16 ertapenem-resistant isolates were meropenem-susceptible; all five meropenem-resistant isolates were also ertapenem-resistant; and all 18 ertapenem-resistant isolates were resistant to ceftazidime or ceftriaxone. In addition, among 16 isolates tested for both imipenem and meropenem, five were imipenem-resistant but meropenem-susceptible. Such patterns can reflect ESBL or AmpC production combined with porin loss, carbapenemases, or methodological artifacts, and no confirmatory testing (MIC determination or carbapenemase detection) was available. The apparent decrease in ertapenem resistance (64.7% to 25.9%) therefore cannot be interpreted.

### 2.7. Multidrug Resistance

Multidrug resistance (MDR) was defined as acquired non-susceptibility to at least one agent in at least three antimicrobial categories, according to Magiorakos et al. [12]. The primary analysis used the core-panel categories in Enterobacterales (aminoglycoside, penicillin [*E. coli* only], second-generation cephalosporin, penicillin plus β-lactamase inhibitor, and folate-pathway inhibitor), because these categories were tested in at least 90% of isolates in both periods. Core-panel MDR was 51.9% (69/133) in 2019–2020 and 38.7% (79/204) in 2024–2025 (difference, −13.2 percentage points; 95% CI, −23.7 to −2.3; *p* = 0.019). The direction was the same in *E. coli* (50.5% vs. 41.3%; *p* = 0.191) and *K. pneumoniae* (56.2% vs. 32.8%; *p* = 0.045) (Table 5). Full-category MDR in Enterobacterales was 68.4% versus 58.3% (*p* = 0.067), and 73.4% versus 65.0% (*p* = 0.134) when at least five eligible categories were required. *P. aeruginosa* (0/6 vs. 0/13) and *Proteus* spp. (0/1 vs. 1/2) are shown descriptively. Pooled four-species MDR (65.0% vs. 54.8%; *p* = 0.062) is a secondary analysis because pooling heterogeneous species and testing panels may be confounded. Excluding the eight children from 2020 did not change the results (core-panel MDR, 51.6% vs. 38.7%; *p* = 0.023).

### 2.8. Adjusted Analyses

Because sex distribution, ultrasound findings, and VUR frequency differed between periods, we estimated odds ratios for 2024–2025 versus 2019–2020 at the isolate (child) level using Firth penalized logistic regression, adjusted for male sex, age ≥ 12 months, and structural anomaly or urolithiasis on ultrasonography (Table 6 and Table S3). Adjustment changed the estimates little. For core-panel agents, no adjusted association remained significant after correction (all *q* ≥ 0.061); the adjusted odds ratio for gentamicin resistance in *K. pneumoniae* was 0.21 (95% CI, 0.07–0.65; *p* = 0.007; *q* = 0.061). Adjusted core-panel MDR in Enterobacterales was lower in 2024–2025 (OR 0.56; 95% CI, 0.35–0.88; *p* = 0.013), as was adjusted full-category MDR (OR 0.58; 95% CI, 0.36–0.94; *p* = 0.027), but not when at least five categories were required (OR 0.61; 95% CI, 0.37–1.03; *p* = 0.064). The *K. pneumoniae* models were based on few events and yielded wide confidence intervals. The years 2019 and 2020 could not be analyzed separately because only eight children were from 2020.

## 3. Discussion

In this single-center study of hospitalized children under five years of age, we compared resistance between 2019–2020 and 2024–2025. Two features limit the interpretation. First, the susceptibility results rely on categorical interpretations recorded by the laboratory (disk diffusion). The same identification and testing methods and the same EUCAST breakpoints version (9.0) were used in both periods, so changes in methods or breakpoints are an unlikely explanation for differences between periods. However, original inhibition-zone diameters and individual quality-control results were not available, so the results could not be verified, and differences may still partly reflect changes in testing practices rather than true changes in resistance. Second, second-line agents were tested selectively, predominantly in cefuroxime-resistant isolates. We therefore restricted the primary analyses to a core panel tested in at least 90% of isolates. In this panel, no resistance proportion differed significantly in *E. coli*, and the nominal decrease in cefuroxime resistance in *K. pneumoniae* did not survive correction. Among exploratory agents, the nominal decreases in resistance to third-generation cephalosporins, cefixime, ertapenem, and amikacin also did not remain significant after correction and cannot be interpreted because of selective testing. Core-panel MDR in Enterobacterales was lower in the second period, whereas full-category MDR was not significantly lower. These findings are best interpreted as center-specific periodic surveillance rather than evidence of a true temporal trend.

*E. coli* was the most frequently isolated pathogen in both periods, consistent with the pediatric UTI literature. In the SENTRY surveillance study of 3511 pediatric uropathogenic *E. coli* isolates in the United States between 2014 and 2023, *E. coli* remained the dominant pathogen in childhood UTI, although susceptibility trends varied by pathogen [3]. Similarly, in a five-year single-center study in Poland, *E. coli* was the primary pathogen, and resistance rates did not follow the same trend across years and antibiotics [4]. A pediatric surveillance study in Iran covering 2005–2016 also showed that the distribution of uropathogens and susceptibility patterns changed over time, indicating that empirical treatment recommendations should not be applied unchanged for long periods [13]. These data suggest that national or international averages alone may not reflect resistance patterns at an individual center. In *E. coli*, the core-panel resistance proportions in 2024–2025 (ampicillin, 72.6%; cefuroxime, 56.0%) were similar to those reported from Turkish pediatric centers since 2020 (ampicillin, 67.4–84.8%; cefuroxime, 44.4–59.1%) and somewhat lower for amoxicillin-clavulanate (37.4% vs. 44.9–72.7%) and TMP-SMX (31.4% vs. 33.3–54.5%) [9,10].

Resistance to third-generation cephalosporins among all *E. coli* isolates was at least 66.3% in 2019–2020 and 55.2% in 2024–2025 (Section 2.6; the difference was not statistically significant), which is higher than the resistance to third-generation cephalosporins or the ESBL proportions reported in recent pediatric surveillance from Korea, Israel, and the United States [3,5,6]. Whether this reflects ESBL production could not be determined because ESBL confirmation was unavailable; the very high proportions among tested isolates (71–90%) largely reflect selective testing. A pediatric cohort study in Taiwan similarly showed that resistance trends varied by antibiotic and period and should be interpreted together with patient characteristics and healthcare-associated exposure [14]. An Istanbul study reported ESBL production in 50% of 66 *E. coli* isolates [10], which is of the same order as our estimate, although ESBL status was not available in our study.

The apparent decreases in resistance to ertapenem, cefixime, and amikacin in *K. pneumoniae* were large but were based on few, selectively tested isolates. Carbapenem results were internally discordant (Section 2.6), and no confirmatory testing was available, so these findings cannot be interpreted as true changes. Carbapenem resistance of 15–23% in *K. pneumoniae* has also been reported from Adıyaman (meropenem, 15.5%; imipenem, 16.4%; ertapenem, 23.4%) [9], so such results are not unique to our center, but they require confirmatory testing. A study of uropathogens during the COVID-19 pandemic in Romania showed that resistance patterns may be influenced by healthcare system burden, antibiotic use, and hospital-associated transmission [15], and opposing patterns may occur at different centers during the same period. Amikacin susceptibility alone is insufficient to justify an empirical treatment recommendation, because treatment effectiveness, drug exposure, and toxicity were not evaluated.

Multidrug resistance depends on which categories are tested. Core-panel MDR, which relies on categories tested in at least 90% of isolates, was lower in the second period (51.9% vs. 38.7%), with a similar direction in *E. coli* and *K. pneumoniae*; the adjusted odds ratio was 0.56 (95% CI, 0.35–0.88). Full-category MDR according to Magiorakos et al. [12] was not significantly lower (68.4% vs. 58.3%; *p* = 0.067), and it was not significantly lower when at least five eligible categories were required (*p* = 0.134). The core panel is a post hoc and limited definition: it omits carbapenems, fluoroquinolones, and extended-spectrum cephalosporins, which are central to empirical therapy, and cefuroxime resistance served only as a proxy for cephalosporin resistance. The MDR result should therefore be regarded as a center-specific surveillance signal that depends on the definition used, and confirmation with a standardized susceptibility-testing panel applied to all isolates is warranted.

Because sex distribution, ultrasound findings, and VUR frequency differed between periods, we examined confounding by case mix using penalized regression adjusted for sex, age, and ultrasound findings. The direction and magnitude of the between-period differences were similar after adjustment, and no antibiotic-specific association in the core panel remained significant after correction. These analyses cannot account for unmeasured factors, such as prior antibiotic exposure, prophylaxis, or changes in testing practices, and the *K. pneumoniae* models were based on few events and yielded wide intervals.

Antibiotic stewardship programs are becoming increasingly important in the treatment of pediatric UTI. A study of an antibiotic stewardship program among hospitalized children reported that recommendations adapted to local microbiological surveillance could improve prescribing adherence [16]. However, we did not directly examine antibiotic consumption, changes in prescribing practices, prophylaxis use, or adherence to a stewardship program. Therefore, we cannot attribute the lower resistance proportions observed in the second period to stewardship practices. The most reliable clinical implication of our findings is that empirical treatment protocols should be reviewed regularly using up-to-date, center-specific susceptibility data.

The COVID-19 pandemic separates the two study periods. Almost all children in the first period (185 of 193) were hospitalized in 2019, before the pandemic, so a pandemic effect within the first period is unlikely, whereas the second period follows it. During the pandemic, healthcare-seeking behavior in children, hospitalization thresholds, community antibiotic consumption, and infection circulation changed, and recent studies indicate that its effect on uropathogen resistance is not unidirectional [15,17]. Differences between the 2019–2020 and 2024–2025 periods should therefore not be interpreted solely as natural changes in resistance; pandemic-related changes in healthcare utilization and patient selection are possible explanations.

In our study, the proportion of male patients and the proportion of structural anomalies or urolithiasis on ultrasonography increased in the second period, while the VUR rate decreased. Sex, age, urinary tract anomalies, prior antibiotic exposure, and healthcare-associated infections may be associated with resistant uropathogens. Consequently, crude resistance comparisons between the periods may have been influenced by these differences in patient profiles. Differences in imaging findings may also reflect changes in patient mix and in the timing of imaging between the two periods. Future studies with larger samples and data on prior antibiotic exposure and prophylaxis are needed to address residual confounding.

This study has several limitations. First, the single-center, retrospective design limits generalizability. The findings apply only to hospitalized children under five years of age at this center; outpatient children, children with recurrent UTI, and broader regional surveillance data were not included, and the results should not be generalized to pediatric UTI in Türkiye as a whole. A first episode was defined from the history provided by the family and recorded at admission, so undocumented previous episodes cannot be fully excluded. Second, second-line agents were not tested with the same frequency across all isolates; they were tested predominantly in cefuroxime-resistant isolates, so their resistance proportions are biased and not comparable between periods, and sample sizes were small for several species–antibiotic comparisons. The laboratory reported second-line agents in a cascade manner, but the exact reporting rules were not available. Third, the study files did not contain the original inhibition-zone diameters or individual quality-control results, so the recorded categorical results could not be verified or reinterpreted. The laboratory used the same identification and testing methods and the same EUCAST breakpoints version (9.0) in both periods, which makes changes in methods or breakpoints an unlikely explanation for between-period differences; however, changes in testing panels and reporting practices cannot be excluded and may affect the main temporal comparison. Intermediate results were classified as susceptible in both periods. This is consistent across periods and follows the EUCAST definition of “I” as “susceptible, increased exposure,” introduced in 2019 [18], rather than the earlier “intermediate” category; non-susceptibility and MDR may therefore be lower than if intermediate results had been counted as non-susceptible [12]. Fourth, carbapenem results were discordant, and no confirmatory testing for carbapenemases or ESBL/AmpC production was available. Fifth, the core panel and primary outcomes were defined post hoc during revision, and the Benjamini–Hochberg families (core and exploratory) were defined by agent role; the results should therefore be considered exploratory. Sixth, data on prior antibiotic use, prophylaxis, pre-culture treatment, healthcare-associated infection status, and antibiotic consumption could not be evaluated. Seventh, urine was collected by clean-catch midstream sampling in toilet-trained children and by sterile bag in the others, but the collection method was not recorded for individual children. Because the median age was 4 months and 73.7% of children were younger than 24 months, most specimens were probably bag samples, which have higher contamination rates than clean-catch, catheter, or suprapubic specimens [19,20]. Some isolates may therefore reflect periurethral colonization rather than infection, which could bias pathogen distribution and resistance estimates. Contaminated cultures were analyzed separately (22.6% in children younger than 24 months vs. 20.6% in older children), and the age distribution was similar between periods (76.2% vs. 72.1% younger than 24 months), but residual misclassification cannot be excluded. Eighth, ESBL phenotype or genotype data were not available, so the contribution of ESBL production to cephalosporin resistance could not be assessed. VCUG and DMSA scintigraphy were performed about 3 weeks after the infection in 2019–2020 but about 12 weeks after it in 2024–2025 because of a change in the unit protocol. This may explain the lower frequency of vesicoureteral reflux in 2024–2025 (0 of 305 children vs. 12 of 193) and of abnormal DMSA findings, for example through spontaneous resolution of transient reflux or of acute scintigraphic changes; imaging findings are therefore not comparable between periods. Ultrasonography was performed during the acute infection, and hydronephrosis was not graded, although transient dilatation is common during acute UTI. Finally, differences in sex distribution and imaging findings between periods may introduce confounding, which the adjusted analyses addressed only for the measured covariates. Despite these limitations, the study provides updated center-specific surveillance data on pediatric UTI isolates, including species-level resistance and MDR patterns, and illustrates how selective testing can bias resistance surveillance.

## 4. Materials and Methods

### 4.1. Study Design and Population

This was a single-center, retrospective, repeated cross-sectional study of two study periods. We included children under five years of age who were hospitalized in the Department of Pediatrics of Kartal Dr. Lütfi Kırdar City Hospital between 1 January 2019 and 31 December 2020 and between 1 January 2024 and 31 December 2025 with a clinical diagnosis of a first UTI episode. We excluded children with recurrent UTI, chronic kidney disease, immunodeficiency, or major non-urological congenital anomalies; congenital anomalies of the kidney and urinary tract were not exclusion criteria. A total of 498 patients were included: 193 from 2019–2020 and 305 from 2024–2025. A first episode was defined as the absence of a previous UTI in the history obtained from the family and recorded in the medical record at admission, including episodes treated at other institutions; undocumented episodes cannot be fully excluded.

Diagnostic definition. UTI was diagnosed in children with fever ≥ 38 °C or urinary/systemic symptoms and pyuria (≥5 leukocytes per high-power field in centrifuged urine, ≥10 leukocytes/µL, or positive leukocyte esterase/nitrite). Urine was collected by clean-catch midstream sampling in toilet-trained children and by sterile adhesive collection bag in children who were not toilet-trained (routine practice of the unit; the collection method was not recorded for individual children). Urethral catheterization was not used. Clean-catch midstream urine has good diagnostic accuracy in toilet-trained children [21], whereas bag specimens are more prone to contamination (43.9% vs. 14.3% for clean-catch specimens in a Turkish pediatric hospital [19]; see also [20]). Growth of a single organism at ≥10^4^ CFU/mL was considered significant, consistent with the EAU/ESPU criterion for symptomatic children [21]. Cultures with mixed growth were reported by the laboratory as contaminated, consistent with the EAU/ESPU recommendation that mixed cultures indicate contamination [22].

Units of analysis. Each of the 498 records corresponds to one hospitalization with one index urine culture. Throughout the manuscript, “patients” and “children” refer to the 498 hospitalized children, “cultures” to their index urine cultures, and “isolates” to the 359 cultures with growth of one of the four target species; “growth present” denotes growth of one of these species. Of 549 children assessed, 32 were excluded because of recurrent UTI, chronic kidney disease, immunodeficiency, or major non-urological congenital anomalies. Of the remaining 517 children with a study-period record, 16 without a recorded culture result and 3 with growth of other organisms (Staphylococcus aureus, *n* = 2; Serratia marcescens, *n* = 1) were not included (Figure 1). No growth of other organisms (for example, Enterococcus, Enterobacter, Citrobacter, or Morganella) was recorded.

This study was approved by the Scientific Research Ethics Committee of Kartal Dr. Lütfi Kırdar City Hospital (Decision No.: 2026/010.99/27/23; Date: 29 April 2026). The study was conducted in accordance with the Declaration of Helsinki.

### 4.2. Data Collection

We retrospectively retrieved patients’ demographic characteristics (age, sex), length of hospital stay, and findings from urinary system ultrasonography (USG), voiding cystourethrography (VCUG), and dimercaptosuccinic acid (DMSA) scintigraphy from the hospital information management system. UTI status was based on the clinical diagnosis recorded for the index hospitalization. Urine culture results were evaluated separately and categorized as no growth, contaminated, or growth of a pathogen. Antimicrobial resistance analyses were restricted to the 359 culture-positive isolates of the four target species (*Proteus* spp., *Pseudomonas aeruginosa*, *Klebsiella pneumoniae*, and *Escherichia coli*).

A procedure was considered performed when its result was recorded in the hospital information system, and the percentages in Table 1 use the number of children with a recorded result as the denominator. VCUG and DMSA scintigraphy results were available for all 498 children and ultrasonography results for 496 children; two children in 2019–2020 had no recorded ultrasonography result and were excluded from the ultrasonography percentages. Imaging results were recorded in the hospital information system as short free-text entries and were categorized for this study. Ultrasonography findings were classified as structural anomaly (hydronephrosis, cyst, atrophy, ectopic kidney, multicystic dysplastic kidney, or renal agenesis), urolithiasis, or infection-related finding (cystitis or pyelonephritis); infection-related findings were not counted as anomalies. Ultrasonography was performed during the acute infection. According to the unit protocol, VCUG and DMSA scintigraphy were performed in all children approximately 3 weeks after the acute infection in 2019–2020 and approximately 12 weeks after it in 2024–2025 (a change in protocol), and the imaging results were verified against the medical records. Hydronephrosis was not graded.

### 4.3. Microbiological Evaluation

Urine culture processing, species identification, and antimicrobial susceptibility testing were performed as part of routine clinical microbiology practice in the hospital laboratory. The same methods were used in both study periods. Species were identified by MALDI-TOF mass spectrometry (MALDI Biotyper; Bruker Daltonics, Bremen, Germany), which also differentiated *K. pneumoniae* from other *Klebsiella* species. Antimicrobial susceptibility was determined by disk diffusion (Kirby–Bauer) using antibiotic disks (Bioanalyse, Ankara, Türkiye), with *E. coli* ATCC 25922 (American Type Culture Collection, Manassas, VA, USA) as the quality-control strain, and the laboratory interpreted the results according to EUCAST clinical breakpoints, version 9.0. Second-line agents were reported in a cascade (selective) manner according to the laboratory’s routine practice. For this retrospective study, we used the categorical susceptibility interpretations recorded in the hospital microbiology records. The study files did not contain the underlying inhibition-zone diameters or the results of individual quality-control runs; consequently, the recorded categorical results could not be verified or reinterpreted. *Proteus* isolates were recorded at genus level in the study files.

The following antimicrobial results were recorded, when available, for Enterobacterales and *P. aeruginosa*: piperacillin-tazobactam, meropenem, ertapenem, ciprofloxacin, imipenem, ceftazidime, ampicillin, cefixime, ceftriaxone, cefuroxime, amikacin, gentamicin, amoxicillin-clavulanate, TMP-SMX, cefepime, and ampicillin-sulbactam. We did not analyze nitrofurantoin and fosfomycin (oral agents indicated for uncomplicated UTI; EUCAST oral fosfomycin breakpoints apply to *E. coli* only), colistin (EUCAST interpretation requires broth microdilution), or agent–organism combinations without applicable EUCAST breakpoints, and we omitted rows without tested isolates. Cefixime breakpoints apply to uncomplicated UTI only, so cefixime results should be interpreted with caution. Nitrofurantoin and oral fosfomycin reach therapeutic concentrations in urine only and are not used for febrile or upper-tract UTI, whereas cefixime is a systemic oral cephalosporin used as step-down therapy in children and was therefore retained as an exploratory agent. Ampicillin resistance in *K. pneumoniae* reflects intrinsic resistance and was not analyzed as acquired resistance.

MDR was defined as acquired non-susceptibility to at least one agent in at least three antimicrobial categories, according to Magiorakos et al. [12]. For Enterobacterales, the eligible categories represented in the dataset were aminoglycosides, antipseudomonal penicillins plus β-lactamase inhibitors, carbapenems, non-extended-spectrum cephalosporins, extended-spectrum cephalosporins, fluoroquinolones, folate-pathway inhibitors, penicillins (not for *K. pneumoniae* or *Proteus* spp.), and penicillins plus β-lactamase inhibitors. For *P. aeruginosa*, the categories were aminoglycosides, carbapenems, antipseudomonal cephalosporins, fluoroquinolones, and piperacillin-tazobactam. Cefixime and nitrofurantoin were not counted, and the fosfomycin and polymyxin categories were excluded because their results were not analyzed. Species-specific intrinsic resistance was excluded from category counting; because *Proteus* isolates were recorded only at genus level, the penicillin category was conservatively excluded for *Proteus* spp. All 359 isolates had results in at least three eligible categories. Core-panel MDR used the following categories in Enterobacterales, which were tested in at least 90% of isolates in both periods: aminoglycoside (gentamicin), penicillin (ampicillin; *E. coli* only), second-generation cephalosporin (cefuroxime), penicillin plus β-lactamase inhibitor (amoxicillin-clavulanate), and folate-pathway inhibitor (TMP-SMX).

Handling of intermediate susceptibility. In the extracted dataset, results were recorded only as susceptible or resistant. Intermediate (“I”) results were included in the susceptible category in both study periods, consistent with the current EUCAST definition of “I” as “susceptible, increased exposure” [18]. “Resistant” in this manuscript therefore refers only to isolates recorded as resistant, and non-susceptibility in the MDR definition [12] corresponded to the recorded resistant category. Because intermediate isolates were classified as susceptible, MDR proportions may be lower than if intermediate results had been counted as non-susceptible, as in the original definition [12].

### 4.4. Statistical Analysis

Data were analyzed using IBM SPSS Statistics, version 31 (IBM Corp., Armonk, NY, USA). Categorical variables are presented as counts and percentages [*n* (%)], and numerical variables that did not follow a normal distribution as medians (first quartile–third quartile). All tests were two-sided. For demographic, clinical, and imaging comparisons of 2 × 2 tables, we used Pearson’s chi-square test when all expected frequencies were ≥25, the Yates-corrected chi-square test when the smallest expected frequency was between 5 and 25, and Fisher’s exact test when it was <5. We used the Fisher–Freeman–Halton exact test for larger tables with sparse cells and the Mann–Whitney U test for numerical variables. All between-period comparisons of resistance and MDR proportions used Fisher’s exact test and were calculated only when at least 10 isolates were tested in both periods. Proportions are presented with Wilson 95% confidence intervals, and between-period differences (percentage points) with Newcombe 95% confidence intervals. Unadjusted *p*-values < 0.05 were considered nominally significant.

Primary outcomes were defined during revision, after review of testing coverage (a post hoc decision), as resistance to a core panel of agents with applicable EUCAST breakpoints tested in at least 90% of isolates of a species in both periods (nitrofurantoin, tested in more than 90% of *E. coli* isolates, was not included because its breakpoints apply to uncomplicated UTI) (*E. coli*: ampicillin, cefuroxime, gentamicin, amoxicillin-clavulanate, and TMP-SMX; *K. pneumoniae*: cefuroxime, gentamicin, amoxicillin-clavulanate, and TMP-SMX) and core-panel MDR in Enterobacterales. All other agents were exploratory. Testing coverage was defined as the proportion of isolates of a species tested for an agent in a period (Table S2), and selective testing was assessed by comparing cefuroxime resistance between isolates tested and not tested for each agent (Table S4). We applied the Benjamini–Hochberg procedure separately to the primary (core) family (9 comparisons) and the exploratory family (16 comparisons), each combining *E. coli* and *K. pneumoniae*, as a false-discovery-rate analysis [23]; *q*-values < 0.05 were considered significant (Tables S1 and S3). To assess confounding by case mix, we estimated unadjusted and adjusted odds ratios for 2024–2025 versus 2019–2020 at the isolate (child) level using Firth penalized logistic regression [24,25], adjusting for male sex, age ≥ 12 months, and structural anomaly or urolithiasis on ultrasonography (and for species in pooled models). Vesicoureteral reflux was not included because no case occurred in 2024–2025, and two isolates with missing ultrasonography results were excluded from the adjusted models. We used Firth’s method because of sparse data and separation (for example, no MDR isolates among *P. aeruginosa*); 95% confidence intervals are Wald intervals. Sensitivity analyses restricted MDR to isolates with results in at least four and at least five eligible categories and excluded the eight children from 2020. The penalized regression, the Benjamini–Hochberg calculations for the adjusted analyses, and the confidence intervals were computed in Python (version 3.12; Python Software Foundation, Wilmington, DE, USA) using NumPy (version 2.1), SciPy (version 1.15), and statsmodels (version 0.14). The study is reported according to the STROBE statement [26]; the checklist is provided as Supplementary Material.

## 5. Conclusions

Among hospitalized children under five years of age at a single center, second-line agents were tested selectively, predominantly in cefuroxime-resistant isolates, so resistance proportions for third-generation cephalosporins, ertapenem, and amikacin are biased, and their apparent decreases cannot be interpreted. In a core panel tested in at least 90% of isolates, no resistance proportion differed significantly in *E. coli*, and the nominal decrease in cefuroxime resistance in *K. pneumoniae* did not survive correction. Core-panel MDR in Enterobacterales was lower in 2024–2025 (51.9% vs. 38.7%; *p* = 0.019), whereas full-category MDR was not significantly lower (68.4% vs. 58.3%; *p* = 0.067). Although the same EUCAST breakpoints version was applied in both periods, testing coverage differed, and the results could not be verified; these differences may therefore partly reflect changes in testing and reporting rather than true changes in resistance. Resistance to ampicillin (72.6%) and cefuroxime (56.0%) remained high in *E. coli* in 2024–2025. The findings are specific to hospitalized children under five years of age at this center, do not include outpatients or recurrent UTI, and should not be generalized to pediatric UTI in Türkiye. They do not support treatment recommendations beyond regular review of local empirical protocols with standardized, complete susceptibility testing.

## Figures and Tables

**Table 1:** Comparison of Patients’ Demographic, Clinical, and Imaging Characteristics by Study Period.

Variable	2019–2020 (*n* = 193)	2024–2025 (*n* = 305)	Test Statistic	*p*-Value
Sex, *n* (%)				
Male	73 (37.8) a	164 (53.8) b	12.051	<0.001 ‡
Female	120 (62.2) a	141 (46.2) b		
Age, months, median (Q1–Q3)	5 (2–23)	4 (2–30)	0.392	0.695 &
VCUG, *n* (%) (*n* = 193/*n* = 305)				
Normal	181 (93.8) a	305 (100.0) b	–	<0.001 ŧ
VUR	12 (6.2) a	0 (0.0) b		
Length of stay, days, median (Q1–Q3)	10 (7–10)	7 (7–10)	0.826	0.409 &
DMSA, *n* (%) (*n* = 193/*n* = 305)				
Normal	172 (89.1)	284 (93.1)	1.954	0.162 Φ
Abnormal	21 (10.9)	21 (6.9)		
Abnormal DMSA finding, *n* (%) (*n* = 21/*n* = 21)				
Atrophy	21 (100.0)	18 (85.7)		
Ectopic kidney	0 (0.0)	1 (4.8)	–	0.232 ¥
Right renal agenesis	0 (0.0)	2 (9.5)		
Ultrasound, *n* (%) (*n* = 191/*n* = 305)				
Normal	168 (88.0)	237 (77.7)		
Structural anomaly or urolithiasis	23 (12.0) a	65 (21.3) b	6.915	0.009 ‡
Infection-related finding (cystitis, pyelonephritis)	0 (0.0)	3 (1.0)		
Structural anomaly or urolithiasis, *n* (%) (*n* = 23/*n* = 65)				
Hydronephrosis	17 (73.9)	40 (61.5)		
Stone	2 (8.7)	17 (26.2)		
Cyst (including renal cyst)	1 (4.3)	4 (6.2)		
Atrophy	2 (8.7)	0 (0.0)		
Ectopic kidney	0 (0.0)	1 (1.5)		
Multicystic dysplastic kidney	1 (4.3)	0 (0.0)		
Renal agenesis (bilateral/unilateral)	0 (0.0)	3 (4.6)		
Urine culture growth, *n* (%)				
No growth	16 (8.3)	13 (4.3)		
Contaminated	37 (19.2)	73 (23.9)	4.516	0.105 ‡
Growth present	140 (72.5)	219 (71.8)		
Isolated pathogen, *n* (%) (*n* = 140/*n* = 219)				
*Proteus* spp.	1 (0.7)	2 (0.9)		
*Pseudomonas aeruginosa*	6 (4.3)	13 (5.9)	–	0.601 ¥
*Klebsiella pneumoniae*	32 (22.9)	61 (27.9)		
*Escherichia coli*	101 (72.1)	143 (65.3)		

*n*: number of patients; %: column percentage; numerical variables are presented as median (first quartile–third quartile). ‡ Pearson’s chi-square test; ¥ Fisher–Freeman–Halton exact test; Φ Yates’ continuity-corrected chi-square test; ŧ Fisher’s exact test; & Mann–Whitney U test. For Mann–Whitney U comparisons, standardized Z values are shown in the Test statistic column. A dash indicates that no separate test statistic is reported for the exact test. Different superscripts (a, b) within the same row indicate a statistically significant difference between groups. Percentages for each imaging modality use the number of children with a recorded result as the denominator (VCUG and DMSA, *n* = 193 and 305; ultrasonography, *n* = 191 and 305). VCUG and DMSA scintigraphy were performed about 3 weeks (2019–2020) and about 12 weeks (2024–2025) after the acute infection; comparisons of these findings between periods should be interpreted with caution. Structural anomaly or urolithiasis versus all other ultrasound results was compared with Pearson’s chi-square test; infection-related findings were not counted as anomalies.

**Table 2:** Antibiotic Susceptibility Results for *Pseudomonas aeruginosa* Isolates by Study Period.

Antibiotic	2019–2020 *n* Tested	2019–2020 Resistant, *n* (%; 95% CI)	2024–2025 *n* Tested	2024–2025 Resistant, *n* (%; 95% CI)
Piperacillin-tazobactam	6	0 (0.0; 0.0–39.0)	13	0 (0.0; 0.0–22.8)
Meropenem	5	0 (0.0; 0.0–43.4)	11	0 (0.0; 0.0–25.9)
Ciprofloxacin	4	0 (0.0; 0.0–49.0)	7	0 (0.0; 0.0–35.4)
Imipenem	3	1 (33.3; 6.1–79.2)	13	0 (0.0; 0.0–22.8)
Ceftazidime	6	0 (0.0; 0.0–39.0)	12	0 (0.0; 0.0–24.3)
Amikacin	3	0 (0.0; 0.0–56.2)	13	0 (0.0; 0.0–22.8)
Cefepime	2	0 (0.0; 0.0–65.8)	13	0 (0.0; 0.0–22.8)

*n* tested: number of isolates tested; CI: Wilson 95% confidence interval. No *p*-values are given because of the small numbers. Colistin was not analyzed because EUCAST interpretation requires broth microdilution.

**Table 3:** Antibiotic Susceptibility Results for *Klebsiella pneumoniae* Isolates by Study Period.

Antibiotic	2019–2020 *n* Tested	2019–2020 Resistant, *n* (%; 95% CI)	2024–2025 *n* Tested	2024–2025 Resistant, *n* (%; 95% CI)	Difference, pp (95% CI)	Fisher *p*
Core panel (tested in ≥90% of isolates in both periods)						
Ampicillin (intrinsic resistance)	31	31 (100.0; 89.0–100.0)	61	61 (100.0; 94.1–100.0)	—	—
Cefuroxime	32	23 (71.9; 54.6–84.4)	59	29 (49.2; 36.8–61.6)	−22.7 (−40.3 to −1.5)	0.047
Gentamicin	32	13 (40.6; 25.5–57.7)	60	13 (21.7; 13.1–33.6)	−19.0 (−38.1 to +0.3)	0.087
Amoxicillin-clavulanate	32	22 (68.8; 51.4–82.0)	58	35 (60.3; 47.5–71.9)	−8.4 (−26.9 to +12.4)	0.497
TMP-SMX	31	14 (45.2; 29.2–62.2)	55	23 (41.8; 29.7–55.0)	−3.3 (−24.3 to +17.4)	0.823
Exploratory agents (selective testing; see Table S4)						
Piperacillin-tazobactam	24	17 (70.8; 50.8–85.1)	33	27 (81.8; 65.6–91.4)	+11.0 (−10.6 to +33.2)	0.357
Meropenem †	11	1 (9.1; 1.6–37.7)	23	4 (17.4; 7.0–37.1)	+8.3 (−22.2 to +29.4)	1.000
Ertapenem †	17	11 (64.7; 41.3–82.7)	27	7 (25.9; 13.2–44.7)	−38.8 (−60.8 to −8.8)	0.015
Ciprofloxacin †	5	3 (60.0; 23.1–88.2)	35	17 (48.6; 33.0–64.4)	−11.4 (−43.7 to +28.8)	—
Imipenem †	12	5 (41.7; 19.3–68.0)	5	0 (0.0; 0.0–43.4)	−41.7 (−68.0 to +7.2)	—
Ceftazidime	23	22 (95.7; 79.0–99.2)	33	25 (75.8; 59.0–87.2)	−19.9 (−37.1 to +0.3)	0.067
Ceftriaxone	24	22 (91.7; 74.2–97.7)	36	26 (72.2; 56.0–84.2)	−19.4 (−36.7 to +1.7)	0.100
Cefixime ‡	25	20 (80.0; 60.9–91.1)	51	25 (49.0; 35.9–62.3)	−31.0 (−48.2 to −7.7)	0.013
Amikacin †	15	6 (40.0; 19.8–64.3)	21	2 (9.5; 2.7–28.9)	−30.5 (−55.7 to −2.5)	0.046
Cefepime †	3	2 (66.7; 20.8–93.9)	25	14 (56.0; 37.1–73.3)	−10.7 (−43.8 to +38.4)	—

*n* tested: number of isolates tested; CI: Wilson 95% confidence interval; pp: percentage points (2024–2025 minus 2019–2020; Newcombe 95% CI). *p*: two-sided Fisher’s exact test, calculated only when at least 10 isolates were tested in both periods. Core panel: agents with applicable EUCAST breakpoints tested in at least 90% of isolates in both periods (post hoc definition; nitrofurantoin was not included because its breakpoints apply to uncomplicated UTI). † Tested in fewer than 50% of isolates in at least one period (Table S2). ‡ EUCAST cefixime breakpoints apply to uncomplicated UTI only; interpret with caution. Ampicillin resistance in *K. pneumoniae* is intrinsic and was not analyzed as acquired resistance. Nitrofurantoin, fosfomycin, and colistin were not analyzed (oral agents indicated for uncomplicated UTI, oral fosfomycin breakpoints apply to *E. coli* only, and colistin requires broth microdilution). Benjamini–Hochberg *q*-values are given in Table S1; no *q*-value was <0.05.

**Table 4:** Antibiotic Susceptibility Results for *Escherichia coli* Isolates by Study Period.

Antibiotic	2019–2020 *n* Tested	2019–2020 Resistant, *n* (%; 95% CI)	2024–2025 *n* Tested	2024–2025 Resistant, *n* (%; 95% CI)	Difference, pp (95% CI)	Fisher *p*
Core panel (tested in ≥90% of isolates in both periods)						
Ampicillin	96	78 (81.2; 72.3–87.8)	135	98 (72.6; 64.5–79.4)	−8.7 (−19.1 to +2.6)	0.158
Cefuroxime	100	66 (66.0; 56.3–74.5)	141	79 (56.0; 47.8–64.0)	−10.0 (−21.8 to +2.6)	0.142
Gentamicin	98	9 (9.2; 4.9–16.5)	140	14 (10.0; 6.1–16.1)	+0.8 (−7.5 to +8.3)	1.000
Amoxicillin-clavulanate	95	43 (45.3; 35.6–55.3)	131	49 (37.4; 29.6–45.9)	−7.9 (−20.5 to +5.0)	0.273
TMP-SMX	98	34 (34.7; 26.0–44.5)	137	43 (31.4; 24.2–39.6)	−3.3 (−15.5 to +8.6)	0.673
Exploratory agents (selective testing; see Table S4)						
Piperacillin-tazobactam †	44	10 (22.7; 12.8–37.0)	48	16 (33.3; 21.7–47.5)	+10.6 (−7.8 to +27.8)	0.354
Meropenem †	19	0 (0.0; 0.0–16.8)	23	1 (4.3; 0.8–21.0)	+4.3 (−12.8 to +21.0)	1.000
Ertapenem †	35	2 (5.7; 1.6–18.6)	38	5 (13.2; 5.8–27.3)	+7.4 (−7.4 to +22.2)	0.432
Ciprofloxacin †	26	9 (34.6; 19.4–53.8)	73	20 (27.4; 18.5–38.6)	−7.2 (−28.4 to +11.6)	0.616
Imipenem †	11	1 (9.1; 1.6–37.7)	2	0 (0.0; 0.0–65.8)	−9.1 (−37.7 to +57.1)	—
Ceftazidime	67	60 (89.6; 80.0–94.8)	82	62 (75.6; 65.3–83.6)	−13.9 (−25.5 to −1.5)	0.033
Ceftriaxone	65	58 (89.2; 79.4–94.7)	90	64 (71.1; 61.0–79.5)	−18.1 (−29.6 to −5.2)	0.009
Cefixime ‡	79	63 (79.7; 69.6–87.1)	109	66 (60.6; 51.2–69.2)	−19.2 (−31.1 to −5.9)	0.007
Amikacin †	39	3 (7.7; 2.7–20.3)	45	1 (2.2; 0.4–11.6)	−5.5 (−18.2 to +5.1)	0.333
Cefepime †	12	2 (16.7; 4.7–44.8)	41	16 (39.0; 25.7–54.3)	+22.4 (−8.8 to +41.7)	0.185
Ampicillin-sulbactam †	3	2 (66.7; 20.8–93.9)	4	2 (50.0; 15.0–85.0)	−16.7 (−61.0 to +41.1)	—

Abbreviations as in Table 3. † Tested in fewer than 50% of isolates in at least one period (Table S2). ‡ EUCAST cefixime breakpoints apply to uncomplicated UTI only; interpret with caution. Nitrofurantoin, fosfomycin, and colistin were not analyzed (oral agents indicated for uncomplicated UTI, and colistin requires broth microdilution).

**Table 5:** Multidrug Resistance by Species Group and Study Period.

Group	2019–2020, *n*/*N* (%)	2024–2025, *n*/*N* (%)	Difference, pp (95% CI)	Fisher *p*
Enterobacterales, core-panel MDR (primary)	69/133 (51.9)	79/204 (38.7)	−13.2 (−23.7 to −2.3)	0.019
*E. coli*, core-panel MDR	51/101 (50.5)	59/143 (41.3)	−9.2 (−21.5 to +3.4)	0.191
*K. pneumoniae*, core-panel MDR	18/32 (56.2)	20/61 (32.8)	−23.5 (−42.2 to −2.4)	0.045
Enterobacterales, full-category MDR	91/133 (68.4)	119/204 (58.3)	−10.1 (−20.1 to +0.5)	0.067
*E. coli*, full-category MDR	71/101 (70.3)	88/143 (61.5)	−8.8 (−20.2 to +3.4)	0.174
*K. pneumoniae*, full-category MDR	20/32 (62.5)	31/61 (50.8)	−11.7 (−30.7 to +9.4)	0.381
Enterobacterales, full-category MDR, ≥5 categories tested	91/124 (73.4)	119/183 (65.0)	−8.4 (−18.4 to +2.3)	0.134
All four species, full-category MDR (secondary)	91/140 (65.0)	120/219 (54.8)	−10.2 (−20.1 to +0.2)	0.062
*P. aeruginosa* (descriptive)	0/6 (0.0)	0/13 (0.0)	—	—
*Proteus* spp. (descriptive)	0/1 (0.0)	1/2 (50.0)	—	—

MDR: acquired non-susceptibility to at least one agent in at least three antimicrobial categories; in this dataset, non-susceptibility corresponded to the recorded resistant category because intermediate results were classified as susceptible. Core-panel categories: aminoglycoside (gentamicin), penicillin (ampicillin, *E. coli* only), second-generation cephalosporin (cefuroxime), penicillin plus β-lactamase inhibitor (amoxicillin-clavulanate), and folate-pathway inhibitor (TMP-SMX). Full-category MDR used all species-appropriate categories of Magiorakos et al. [12] with interpretable EUCAST breakpoints; intrinsic resistance was excluded. pp: percentage points (Newcombe 95% CI); *p*: Fisher’s exact test.

**Table 6:** Unadjusted and Adjusted Odds Ratios for Resistance and Multidrug Resistance in 2024–2025 Versus 2019–2020.

Comparison	*n* (Events)	Unadjusted OR (95% CI)	Adjusted OR (95% CI)	Adjusted *p*	BH *q*
*Escherichia coli*—Ampicillin	230 (176)	0.59 (0.31–1.11)	0.55 (0.29–1.05)	0.071	0.211
*Escherichia coli*—Cefuroxime	240 (145)	0.64 (0.38–1.09)	0.63 (0.37–1.08)	0.094	0.211
*Escherichia coli*—Gentamicin	237 (23)	1.07 (0.45–2.54)	1.04 (0.44–2.46)	0.929	0.929
*Escherichia coli*—Amoxicillin-clavulanate	225 (92)	0.71 (0.41–1.22)	0.69 (0.40–1.19)	0.178	0.320
*Escherichia coli*—TMP-SMX	235 (77)	0.86 (0.50–1.49)	0.89 (0.50–1.56)	0.672	0.756
*Klebsiella pneumoniae*—Cefuroxime	90 (51)	0.41 (0.16–1.03)	0.37 (0.13–1.07)	0.066	0.211
*Klebsiella pneumoniae*—Gentamicin	91 (26)	0.39 (0.15–0.99)	0.21 (0.07–0.65)	0.007	0.061
*Klebsiella pneumoniae*—Amoxicillin-clavulanate	89 (57)	0.64 (0.25–1.62)	0.58 (0.21–1.63)	0.300	0.449
*Klebsiella pneumoniae*—TMP-SMX	85 (37)	0.82 (0.34–2.01)	0.69 (0.25–1.91)	0.473	0.608
Enterobacterales, core-panel MDR (primary)	335 (148)	0.57 (0.37–0.89)	0.56 (0.35–0.88)	0.013	—
*E. coli*, core-panel MDR	243 (110)	0.68 (0.40–1.13)	0.70 (0.41–1.18)	0.176	—
*K. pneumoniae*, core-panel MDR	92 (38)	0.36 (0.15–0.88)	0.25 (0.09–0.70)	0.008	—
Enterobacterales, full-category MDR	335 (210)	0.62 (0.39–0.98)	0.58 (0.36–0.94)	0.027	—
Enterobacterales, full-category MDR, ≥5 categories	306 (210)	0.66 (0.40–1.09)	0.61 (0.37–1.03)	0.064	—
All four species, full-category MDR (secondary)	357 (211)	0.63 (0.40–0.98)	0.59 (0.37–0.95)	0.031	—

OR < 1 indicates lower odds in 2024–2025. Firth penalized logistic regression adjusted for male sex, age ≥ 12 months, and structural anomaly or urolithiasis (and for species in the pooled models); 95% CI are Wald intervals. BH *q*: Benjamini–Hochberg *q*-value of the adjusted *p*-value within the core family (Table S3); not calculated for MDR models. All analyses are exploratory. Events: resistant (or MDR) isolates.

## Data Availability

The data presented in this study are available on request from the corresponding author due to institutional privacy restrictions on patient-level clinical data.

## Supplementary Materials

The following supporting information can be downloaded at https://www.mdpi.com/article/10.3390/antibiotics15101031/s1: Table S1: Fisher’s exact *p*-values and Benjamini–Hochberg *q*-values for organism-specific antibiotic resistance comparisons; Table S2: antimicrobial testing coverage by species and study period; Table S3: adjusted odds ratios and Benjamini–Hochberg *q*-values for organism-specific antibiotic resistance comparisons (2024–2025 versus 2019–2020); Table S4: assessment of selective testing: cefuroxime resistance among isolates tested and not tested for each agent; Table S5: estimates of resistance to third-generation cephalosporins (ceftazidime or ceftriaxone) among all isolates; STROBE statement—checklist of items that should be included in reports of cross-sectional studies. Reference [26] is cited in the Supplementary Materials.

## Abbreviations

BH, Benjamini–Hochberg; CFU, colony-forming units; CI, confidence interval; DMSA, dimercaptosuccinic acid; ESBL, extended-spectrum β-lactamase; EUCAST, European Committee on Antimicrobial Susceptibility Testing; FDR, false discovery rate; MDR, multidrug resistance; MIC, minimum inhibitory concentration; OR, odds ratio; pp, percentage points; TMP-SMX, trimethoprim-sulfamethoxazole; UTI, urinary tract infection; VCUG, voiding cystourethrography; VUR, vesicoureteral reflux.
