# Supplementary material for: Antibiotic Resistance in Urine Culture Isolates from Hospitalized Children Under Five Years with Urinary Tract Infection: A Comparison of the 2019–2020 and 2024–2025 Periods
Source: Antibiotics (Basel). 2026 Oct 16;15(10):1031. doi: 10.3390/antibiotics15101031 (PMC13700752; doi:10.3390/antibiotics15101031)
Supplement: Supplementary file 1 [file antibiotics-15-01031-s001.zip › STROBE_checklist.pdf]

STROBE Statement—Checklist of Items That Should Be Included in Reports of Cross-Sectional Studies

Manuscript: Antibiotic Resistance in Urine Culture Isolates from Hospitalized Children Under Five Years with Urinary Tract Infection: A Comparison of the 2019–2020 and 2024–2025 Periods

Item	STROBE recommendation	Reported in the manuscript
1	(a) Indicate the study's design with a commonly used term in the title or the abstract. (b) Provide in the abstract an informative and balanced summary of what was done and what was found	Abstract (Methods: retrospective, repeated cross-sectional study); Abstract (all sections)
2	Explain the scientific background and rationale for the investigation being reported	Introduction (paragraphs 1–4)
3	State specific objectives, including any prespecified hypotheses	Introduction (last paragraph; primary outcomes defined during revision are stated as post hoc)
4	Present key elements of study design early in the paper	Section 4.1
5	Describe the setting, locations, and relevant dates, including periods of recruitment, exposure, follow-up, and data collection	Section 4.1 (single center; 1 January 2019–31 December 2020 and 1 January 2024–31 December 2025)
6	(a) Give the eligibility criteria, and the sources and methods of selection of participants	Section 4.1; Figure 1
7	Clearly define all outcomes, exposures, predictors, potential confounders, and effect modifiers. Give diagnostic criteria, if applicable	Sections 4.1–4.4 (UTI definition, core panel, MDR, covariates)
8*	For each variable of interest, give sources of data and details of methods of assessment (measurement). Describe comparability of assessment methods if there is more than one group	Sections 4.2–4.3 (hospital information system; laboratory methods); Section 2.6 and Limitations (comparability)
9	Describe any efforts to address potential sources of bias	Sections 4.3–4.4 (core panel, selective-testing assessment, sensitivity and adjusted analyses); Section 2.6; Limitations
10	Explain how the study size was arrived at	Section 4.1 and Figure 1 (all eligible children; no sample-size calculation)
11	Explain how quantitative variables were handled in the analyses. If applicable, describe which groupings were chosen and why	Section 4.4 (age ≥12 months; ultrasonography categories)
12	(a) Describe all statistical methods, including those used to control for confounding. (b) Describe any methods used to examine subgroups and interactions. (c) Explain how missing data were addressed. (d) If applicable, describe analytical methods taking account of sampling strategy. (e) Describe any sensitivity analyses	Section 4.4 (a, b, e); Sections 4.2 and 4.4 (c: two missing ultrasonography results excluded); (d) not applicable (all eligible children)
13*	(a) Report numbers of individuals at each stage of the study. (b) Give reasons for non-participation at each stage. (c) Consider use of a flow diagram	Figure 1; Section 2.1; Section 4.1
14*	(a) Give characteristics of study participants and information on exposures and potential confounders. (b) Indicate the number of participants with missing data for each variable of interest	Table 1; Section 2.1; Section 4.2 (denominators for imaging variables)
15*	Report numbers of outcome events or summary measures	Tables 2–5; Sections 2.2–2.7
16	(a) Give unadjusted estimates and, if applicable, confounder-adjusted estimates and their precision (e.g., 95% confidence interval). (b) Report category boundaries when continuous variables were categorized. (c) If relevant, consider translating estimates of relative risk into absolute risk for a meaningful time period	Tables 3–6 and Table S3 (a, c: differences in percentage points with 95% CI); Section 4.4 (b)
17	Report other analyses done (e.g., subgroup analyses, sensitivity analyses)	Sections 2.6–2.8; Tables S1–S4

Item	STROBE recommendation	Reported in the manuscript
18	Summarize key results with reference to study objectives	Discussion (first paragraph)
19	Discuss limitations of the study, taking into account sources of potential bias or imprecision. Discuss both direction and magnitude of any potential bias	Discussion (limitations paragraph)
20	Give a cautious overall interpretation of results considering objectives, limitations, multiplicity of analyses, results from similar studies, and other relevant evidence	Discussion; Conclusions
21	Discuss the generalizability (external validity) of the study results	Discussion (limitations paragraph); Conclusions
22	Give the source of funding and the role of the funders for the present study	Funding statement (no external funding)

** Give information separately for exposed and unexposed groups (here, for the two study periods).*

Reference: von Elm, E.; Altman, D.G.; Egger, M.; Pocock, S.J.; Gøtzsche, P.C.; Vandenbroucke, J.P. Lancet 2007, 370, 1453–1457.
