# Supplementary material for: Antibiotic Resistance in Urine Culture Isolates from Hospitalized Children Under Five Years with Urinary Tract Infection: A Comparison of the 2019–2020 and 2024–2025 Periods
Source: Antibiotics (Basel). 2026 Oct 16;15(10):1031. doi: 10.3390/antibiotics15101031 (PMC13700752; doi:10.3390/antibiotics15101031)
Supplement: Supplementary file 1 [file antibiotics-15-01031-s001.zip › Supplementary_Tables_S1-S5.pdf]

Supplementary Materials

Antibiotic Resistance in Urine Culture Isolates from Hospitalized Children Under Five Years with Urinary Tract Infection: A Comparison of the 2019–2020 and 2024–2025 Periods

Table S1. Fisher's Exact p-Values and Benjamini–Hochberg q-Values for Organism-Specific Antibiotic Resistance Comparisons.

Species	Antibiotic	Group	Fisher p	BH q (within family)
Escherichia coli	Ampicillin	Core	0.158	0.357
Escherichia coli	Cefuroxime	Core	0.142	0.357
Escherichia coli	Gentamicin	Core	1.000	1.000
Escherichia coli	Amoxicillin-clavulanate	Core	0.273	0.491
Escherichia coli	TMP-SMX	Core	0.673	0.865
Escherichia coli	Piperacillin-tazobactam	Exploratory	0.354	0.476
Escherichia coli	Meropenem	Exploratory	1.000	1.000
Escherichia coli	Ertapenem	Exploratory	0.432	0.532
Escherichia coli	Ciprofloxacin	Exploratory	0.616	0.704
Escherichia coli	Ceftazidime	Exploratory	0.033	0.106
Escherichia coli	Ceftriaxone	Exploratory	0.009	0.058
Escherichia coli	Cefixime	Exploratory	0.007	0.058
Escherichia coli	Amikacin	Exploratory	0.333	0.476
Escherichia coli	Cefepime	Exploratory	0.185	0.329
Klebsiella pneumoniae	Cefuroxime	Core	0.047	0.357
Klebsiella pneumoniae	Gentamicin	Core	0.087	0.357
Klebsiella pneumoniae	Amoxicillin-clavulanate	Core	0.497	0.746
Klebsiella pneumoniae	TMP-SMX	Core	0.823	0.925
Klebsiella pneumoniae	Piperacillin-tazobactam	Exploratory	0.357	0.476
Klebsiella pneumoniae	Meropenem	Exploratory	1.000	1.000
Klebsiella pneumoniae	Ertapenem	Exploratory	0.015	0.058
Klebsiella pneumoniae	Ceftazidime	Exploratory	0.067	0.152
Klebsiella pneumoniae	Ceftriaxone	Exploratory	0.100	0.200
Klebsiella pneumoniae	Cefixime	Exploratory	0.013	0.058
Klebsiella pneumoniae	Amikacin	Exploratory	0.046	0.123

BH: Benjamini–Hochberg. The procedure was applied separately to the core family (9 comparisons) and the exploratory family (16 comparisons), each combining E. coli and K. pneumoniae. Only comparisons with at least 10 tested isolates in both periods were included. No q-value was < 0.05.

Table S2. Antimicrobial Testing Coverage by Species and Study Period.

Species	Antibiotic	2019–2020 tested/isolates (%)	2024–2025 tested/isolates (%)	Diff. (pp)	Coverage <50% in either period	Adequate and comparable
Escherichia coli	Ampicillin	96/101 (95.0)	135/143 (94.4)	-0.6	No	Yes
Escherichia coli	Cefuroxime	100/101 (99.0)	141/143 (98.6)	-0.4	No	Yes

Species	Antibiotic	2019–2020 tested/isolates (%)	2024–2025 tested/isolates (%)	Diff. (pp)	Coverage <50% in either period	Adequate and comparable
Escherichia coli	Gentamicin	98/101 (97.0)	140/143 (97.9)	+0.9	No	Yes
Escherichia coli	Amoxicillin-clavulanate	95/101 (94.1)	131/143 (91.6)	-2.5	No	Yes
Escherichia coli	TMP-SMX	98/101 (97.0)	137/143 (95.8)	-1.2	No	Yes
Escherichia coli	Piperacillin-tazobactam	44/101 (43.6)	48/143 (33.6)	-10.0	Yes	No
Escherichia coli	Meropenem	19/101 (18.8)	23/143 (16.1)	-2.7	Yes	No
Escherichia coli	Ertapenem	35/101 (34.7)	38/143 (26.6)	-8.1	Yes	No
Escherichia coli	Ciprofloxacin	26/101 (25.7)	73/143 (51.0)	+25.3	Yes	No
Escherichia coli	Imipenem	11/101 (10.9)	2/143 (1.4)	-9.5	Yes	No
Escherichia coli	Ceftazidime	67/101 (66.3)	82/143 (57.3)	-9.0	No	Yes
Escherichia coli	Ceftriaxone	65/101 (64.4)	90/143 (62.9)	-1.4	No	Yes
Escherichia coli	Cefixime	79/101 (78.2)	109/143 (76.2)	-2.0	No	Yes
Escherichia coli	Amikacin	39/101 (38.6)	45/143 (31.5)	-7.1	Yes	No
Escherichia coli	Cefepime	12/101 (11.9)	41/143 (28.7)	+16.8	Yes	No
Escherichia coli	Ampicillin-sulbactam	3/101 (3.0)	4/143 (2.8)	-0.2	Yes	No
Klebsiella pneumoniae	Cefuroxime	32/32 (100.0)	59/61 (96.7)	-3.3	No	Yes
Klebsiella pneumoniae	Gentamicin	32/32 (100.0)	60/61 (98.4)	-1.6	No	Yes
Klebsiella pneumoniae	Amoxicillin-clavulanate	32/32 (100.0)	58/61 (95.1)	-4.9	No	Yes
Klebsiella pneumoniae	TMP-SMX	31/32 (96.9)	55/61 (90.2)	-6.7	No	Yes
Klebsiella pneumoniae	Piperacillin-tazobactam	24/32 (75.0)	33/61 (54.1)	-20.9	No	No
Klebsiella pneumoniae	Meropenem	11/32 (34.4)	23/61 (37.7)	+3.3	Yes	No
Klebsiella pneumoniae	Ertapenem	17/32 (53.1)	27/61 (44.3)	-8.9	Yes	No
Klebsiella pneumoniae	Ciprofloxacin	5/32 (15.6)	35/61 (57.4)	+41.8	Yes	No
Klebsiella pneumoniae	Imipenem	12/32 (37.5)	5/61 (8.2)	-29.3	Yes	No
Klebsiella pneumoniae	Ceftazidime	23/32 (71.9)	33/61 (54.1)	-17.8	No	No
Klebsiella pneumoniae	Ceftriaxone	24/32 (75.0)	36/61 (59.0)	-16.0	No	No
Klebsiella pneumoniae	Cefixime	25/32 (78.1)	51/61 (83.6)	+5.5	No	Yes
Klebsiella pneumoniae	Amikacin	15/32 (46.9)	21/61 (34.4)	-12.4	Yes	No
Klebsiella pneumoniae	Cefepime	3/32 (9.4)	25/61 (41.0)	+31.6	Yes	No

pp: percentage points. Coverage: number of isolates tested for the agent divided by the number of isolates of the species in that period. Adequate and comparable: coverage ≥50% in both periods and absolute between-period difference ≤10 percentage points (post hoc criterion).

Table S3. Adjusted Odds Ratios and Benjamini–Hochberg q-Values for Organism-Specific Antibiotic Resistance Comparisons (2024–2025 Versus 2019–2020).

Species	Antibiotic	Group	n (events)	Adjusted OR (95% CI)	Adjusted p	BH q
Escherichia coli	Ampicillin	Core	230 (176)	0.55 (0.29–1.05)	0.071	0.211
Escherichia coli	Cefuroxime	Core	240 (145)	0.63 (0.37–1.08)	0.094	0.211
Escherichia coli	Gentamicin	Core	237 (23)	1.04 (0.44–2.46)	0.929	0.929
Escherichia coli	Amoxicillin-clavulanate	Core	225 (92)	0.69 (0.40–1.19)	0.178	0.320
Escherichia coli	TMP-SMX	Core	235 (77)	0.89 (0.50–1.56)	0.672	0.756
Escherichia coli	Piperacillin-tazobactam	Exploratory	92 (26)	1.65 (0.64–4.25)	0.304	0.405
Escherichia coli	Meropenem	Exploratory	42 (1)	4.15 (0.24–72.48)	0.329	0.405
Escherichia coli	Ertapenem	Exploratory	73 (7)	1.78 (0.37–8.54)	0.470	0.501
Escherichia coli	Ciprofloxacin	Exploratory	99 (29)	0.73 (0.28–1.92)	0.521	0.521
Escherichia coli	Ceftazidime	Exploratory	149 (122)	0.33 (0.13–0.85)	0.022	0.088
Escherichia coli	Ceftriaxone	Exploratory	155 (122)	0.33 (0.13–0.81)	0.016	0.088
Escherichia coli	Cefixime	Exploratory	187 (129)	0.37 (0.19–0.74)	0.005	0.079
Escherichia coli	Amikacin	Exploratory	84 (4)	0.40 (0.07–2.39)	0.314	0.405
Escherichia coli	Cefepime	Exploratory	53 (18)	3.74 (0.70–20.09)	0.124	0.242
Klebsiella pneumoniae	Cefuroxime	Core	90 (51)	0.37 (0.13–1.07)	0.066	0.211
Klebsiella pneumoniae	Gentamicin	Core	91 (26)	0.21 (0.07–0.65)	0.007	0.061
Klebsiella pneumoniae	Amoxicillin-clavulanate	Core	89 (57)	0.58 (0.21–1.63)	0.300	0.449
Klebsiella pneumoniae	TMP-SMX	Core	85 (37)	0.69 (0.25–1.91)	0.473	0.608
Klebsiella pneumoniae	Piperacillin-tazobactam	Exploratory	57 (44)	1.81 (0.38–8.63)	0.456	0.501
Klebsiella pneumoniae	Meropenem	Exploratory	34 (5)	32.83 (0.30–3646.43)	0.146	0.242
Klebsiella pneumoniae	Ertapenem	Exploratory	44 (18)	0.21 (0.04–0.99)	0.049	0.112
Klebsiella pneumoniae	Ceftazidime	Exploratory	55 (46)	0.24 (0.04–1.68)	0.151	0.242
Klebsiella pneumoniae	Ceftriaxone	Exploratory	59 (47)	0.16 (0.03–0.88)	0.035	0.096
Klebsiella pneumoniae	Cefixime	Exploratory	75 (44)	0.24 (0.07–0.81)	0.022	0.088
Klebsiella pneumoniae	Amikacin	Exploratory	36 (8)	0.10 (0.01–0.86)	0.036	0.096

OR < 1 indicates lower odds of resistance in 2024–2025. Firth penalized logistic regression adjusted for male sex, age ≥12 months, and structural anomaly or urolithiasis; 95% CI are Wald intervals. BH q: Benjamini–Hochberg q-value within the core and exploratory families. Models based on very few events are unstable and should be interpreted with caution.

Table S4. Assessment of Selective Testing: Cefuroxime Resistance Among Isolates Tested and Not Tested for Each Agent.

Species	Agent	Cefuroxime-resistant among isolates tested for the agent	Cefuroxime-resistant among isolates not tested for the agent	Fisher p
Escherichia coli	Piperacillin-tazobactam	78/89 (88%)	67/152 (44%)	<0.001
Escherichia coli	Meropenem	40/41 (98%)	105/200 (52%)	<0.001
Escherichia coli	Ertapenem	68/72 (94%)	77/169 (46%)	<0.001
Escherichia coli	Ciprofloxacin	72/96 (75%)	73/145 (50%)	<0.001
Escherichia coli	Imipenem	13/13 (100%)	132/228 (58%)	0.002
Escherichia coli	Ceftazidime	138/146 (95%)	7/95 (7%)	<0.001

Species	Agent	Cefuroxime-resistant among isolates tested for the agent	Cefuroxime-resistant among isolates not tested for the agent	Fisher p
Escherichia coli	Ceftriaxone	136/152 (89%)	9/89 (10%)	<0.001
Escherichia coli	Cefixime	121/185 (65%)	24/56 (43%)	0.003
Escherichia coli	Amikacin	60/83 (72%)	85/158 (54%)	0.006
Escherichia coli	Cefepime	44/53 (83%)	101/188 (54%)	<0.001
Escherichia coli	Ampicillin-sulbactam	5/7 (71%)	140/234 (60%)	0.706
Klebsiella pneumoniae	Piperacillin-tazobactam	45/56 (80%)	7/35 (20%)	<0.001
Klebsiella pneumoniae	Meropenem	32/32 (100%)	20/59 (34%)	<0.001
Klebsiella pneumoniae	Ertapenem	40/42 (95%)	12/49 (24%)	<0.001
Klebsiella pneumoniae	Ciprofloxacin	25/38 (66%)	27/53 (51%)	0.199
Klebsiella pneumoniae	Imipenem	17/17 (100%)	35/74 (47%)	<0.001
Klebsiella pneumoniae	Ceftazidime	50/54 (93%)	2/37 (5%)	<0.001
Klebsiella pneumoniae	Ceftriaxone	52/58 (90%)	0/33 (0%)	<0.001
Klebsiella pneumoniae	Cefixime	45/75 (60%)	7/16 (44%)	0.273
Klebsiella pneumoniae	Amikacin	32/35 (91%)	20/56 (36%)	<0.001
Klebsiella pneumoniae	Cefepime	20/28 (71%)	32/63 (51%)	0.072

Cefuroxime (tested in 99% of isolates) was used as a first-line proxy. A higher proportion of cefuroxime resistance among tested than untested isolates indicates that the agent was tested preferentially in resistant isolates.

Table S5. Estimates of Resistance to Third-Generation Cephalosporins (Ceftazidime or Ceftriaxone) Among All Isolates.

Species	Estimate	2019–2020 n/N (%; 95% CI)	2024–2025 n/N (%; 95% CI)	Difference, pp (95% CI); Fisher p
Escherichia coli	Documented resistance (lower bound)	67/101 (66.3; 56.7–74.8)	79/143 (55.2; 47.1–63.2)	-11.1 (-22.9 to +1.4); p = 0.087
Escherichia coli	Plausible upper (untested cefuroxime-resistant isolates also resistant)	67/101 (66.3)	80/143 (55.9)	—
Escherichia coli	Worst-case upper (all untested isolates resistant)	98/101 (97.0)	126/143 (88.1)	—
Klebsiella pneumoniae	Documented resistance (lower bound)	22/32 (68.8; 51.4–82.0)	30/61 (49.2; 37.1–61.4)	-19.6 (-37.6 to +1.6); p = 0.082
Klebsiella pneumoniae	Plausible upper (untested cefuroxime-resistant isolates also resistant)	22/32 (68.8)	30/61 (49.2)	—
Klebsiella pneumoniae	Worst-case upper (all untested isolates resistant)	30/32 (93.8)	52/61 (85.2)	—

Lower bound: isolates with documented ceftazidime or ceftriaxone resistance divided by all isolates of the species (untested isolates assumed susceptible). Plausible upper: additionally counts untested isolates that were cefuroxime-resistant. Worst-case upper: all untested isolates assumed resistant. The difference and p-value refer to the lower bound and are exploratory.
